# Neighbourhood built environment associations with body size in adults: mediating effects of activity and sedentariness in a cross-sectional study of New Zealand adults

**DOI:** 10.1186/s12889-015-2292-2

**Published:** 2015-09-24

**Authors:** Melody Oliver, Karen Witten, Tony Blakely, Karl Parker, Hannah Badland, Grant Schofield, Vivienne Ivory, Jamie Pearce, Suzanne Mavoa, Erica Hinckson, Paul Sweetsur, Robin Kearns

**Affiliations:** Human Potential Centre, Auckland University of Technology, Auckland, New Zealand; SHORE and Whāriki Research Centre, Massey University, Auckland, New Zealand; Department of Public Health, University of Otago, Wellington, New Zealand; McCaughey VicHealth Community Wellbeing Unit, The University of Melbourne, Melbourne, Australia; School of GeoSciences, University of Edinburgh, Edinburgh, Scotland, UK; School of Environment, The University of Auckland, Auckland, New Zealand

**Keywords:** Obesity, Epidemiology, Physical activity, Geographic information systems, Body mass index, Urban design, Walkability, New Zealand

## Abstract

**Background:**

The aim of this study was to determine the associations between body size and built environment walkability variables, as well as the mediating role of physical activity and sedentary behaviours with body size.

**Methods:**

Objective environment, body size (body mass index (BMI), waist circumference (WC)), and sedentary time and physical activity data were collected from a random selection of 2033 adults aged 20–65 years living in 48 neighbourhoods across four New Zealand cities. Multilevel regression models were calculated for each comparison between body size outcome and built environment exposure.

**Results and Discussion:**

Street connectivity and neighborhood destination accessibility were significant predictors of body size (1 SDchange predicted a 1.27 to 1.41 % reduction in BMI and a 1.76 to 2.29 % reduction in WC). Significantrelationships were also observed for streetscape (1 SD change predicted a 1.33 % reduction in BMI) anddwelling density (1 SD change predicted a 1.97 % reduction in BMI). Mediation analyses revealed asignificant mediating effect of physical activity on the relationships between body size and street connectivity and neighbourhood destination accessibility (explaining between 10.4 and 14.6 % of the total effect). No significant mediating effect of sedentary behaviour was found. Findings from this cross-sectional study of a random selection of New Zealand adults are consistent with international research. Findings are limited to individual environment features only; conclusions cannot be drawn about the cumulative and combined effect of individual features on outcomes.

**Conclusions:**

Built environment features were associated with body size in the expected directions. Objectively-assessed physical activity mediated observed built environment-body size relationships.

## Background

The global obesity epidemic poses one of the greatest challenges in modern public health [1]. Contributors to obesity are mutlifaceted and complex, with behavioural (nutrition, physical activity), metabolic, and environmental factors implicated [2, 3]. Increased understanding of factors associated with obesity at the population level is fundamental to efficacious intervention development and implementation.

Escalating interest in the role of built environments (**BEs**) as being obesogenic, or obesity promoting, has been demonstrated in the last decade [1, 4, 5]. Emerging evidence suggests relationships between obesity and various BE attributes in adults [2, 4, 6–11]; however, to what degree the BE is associated with obesity, and the mechanisms by which this occurs, are yet to be fully determined [2]. Theories suggest direct pathways between the BE and dietary and physical activity behaviours, with subsequent impacts on body size (concurrently acknowledging individual and social environment factors that may influence these relationships) [4, 12, 13]. Stronger and more consistent relationships have been found between BE characteristics and physical activity than for food environments [14, 15]. For example, cross-sectional (predominantly US) research has shown clear relationships between BE features and physical activity behaviours (particularly walking) in adults [8, 16, 17].

The magnitude of the associations between environmental features and physical activity can be considerable - earlier work in the Understanding the Relationship Between Activity and Neighbourhoods (**URBAN**) study in New Zealand demonstrated moderate population-wide effects of BE features with self-reported and objectively-assessed physical activity in adults [18]. A one standard deviation (**SD**) higher destination access, street connectivity, and dwelling density was associated with increased odds of any self-reported physical activity via active transport, walking, or leisure-time activities (ranges in effect sizes from 21 to 44 %), and 7 % higher objectively-assessed (via accelerometer) physical activity levels.

Investigating these relationships poses a number of specific challenges, including inconsistencies in conceptual underpinnings, delimitation of geographical boundaries, operationalization of neighbourhoods (e.g., residential and non-residential spaces), and measures of BE features [2, 4, 10] across studies. Within-study homogeneity in demography and BE attributes has further limited our understanding of these relationships [4, 19]. It is also not clear how much of a contribution preferences for living in more walkable neighbourhoods and ‘self-selection’ into preferred neighbourhoods may have in explaining the observed association of BE with physical activity and body size; the extent of such confounding bias is not well quantified or understood in existing research [7, 9, 13, 20–22]. The likely collinearity of BE features also makes it difficult to isolate the effects of individual BE attributes on outcomes of interest. With regard to the behaviours and outcomes, a majority of studies have relied on participants’ self-reported physical activity [23] and height and weight (to calculate the dependent variable, BMI) [19, 23, 24] respectively; measures known to be limited by social desirability bias [25, 26]. BMI has been consistently employed as the dependent outcome. However, this measure is only a proxy for body fatness and differential health risks may be found across individuals of differing ethnicities. Furthermore, alternative proxy measures – WC in particular - have been shown to have stronger associations than BMI with health status and chronic conditions in adults [27].

It is highly likely that physical activity mediates any association between BE features and body size, but few studies have considered this relationship [7, 23, 28–32]. Such examinations are important to test the theoretical coherence of the association between the BE and body size; that is, if physical activity does not contribute somewhat to this relationship, it is possible that systematic error, most notably confounding, may exist. Furthermore, little is known of the potential mediating effect of sedentary behaviour on this relationship. A clear pathway exists between sedentary time (especially prolonged sitting) and body size [33] and evidence suggests a link between the BE and sedentary time (particularly via sitting during motorised transport) [34]. Thus it is possible that any observed relationship between BE features and body size may be mediated by sedentary time.

In this paper we extend on earlier research through analysis of objective measures of body size (BMI, WC) and physical activity and sedentary behaviour (accelerometry) collected from a random selection of participants living within urban neighbourhoods in New Zealand, purposively selected to maximise area-level variability in walkability. Building on our earlier investigations in the URBAN Study [18], we examine the effect of objectively assessed physical activity and sedentary behaviours on the association between commonly-assessed BE features conceptualised as being associated with physical activity and body size. Hypotheses were that body size would be negatively associated with individual BE features under investigation, and that objectively-assessed physical activity (by decreasing body size) and sedentary behaviours (by increasing body size) would mediate this relationship.

## Methods

Data were drawn from the URBAN Study, for which the methods have been previously reported [35]. This cross-sectional observational study was conducted in four New Zealand cities (Waitakere and North Shore in the Auckland region, Christchurch, and Wellington) between April 2008 and September 2010 (total population = 920,000). The URBAN Study is the New Zealand arm of the multi-country International Physical Activity and Environment Network (IPEN); involving replicate protocols across 12 member countries for assessment of dependent and independent variables [36]. Ethical approval to conduct the research was granted by the host institutions’ ethics committees (AUTEC 07/126; MUHECN 07/045). All participants provided informed written consent to participate.

### Neighbourhood and participant selection

A walkability index was calculated for every meshblock (the smallest geographic census area unit in New Zealand; approximating to 100 people) across the four cities (*n* = 7,614). The index was calculated using geographic information systems (GIS)-derived spatial measures of dwelling density, land-use mix, street connectivity, and retail floor area ratio as described elsewhere [18, 37]. Summary scores (average of the mesh-block level walkability z-score values) were calculated for each neighbourhood and neighbourhoods were partitioned into walkability tertiles (low/medium/high). Meshblocks in the highest and lowest tertiles of derived walkability scores were deemed eligible for selection.

Distribution of usual Māori residents domiciled within each mesh-block within the four cities was estimated by using 2006 census data [38]. Following the walkability index procedures, the mesh-block Māori population density was classified into deciles and recoded into values from 1 (1st decile) to 10 (10th decile) for each city. Māori comprise 14.6 % of the resident population. They are the second largest ethnic group (after New Zealand Pākehā/ European) in New Zealand [39].

Study neighbourhoods were defined as clusters of five contiguous meshblocks within the same strata (i.e., high or low walkability, and high or low Māori population). This was a deliberate approach to ensure geographical spread and facilitate ethnic diversity within each region. Overall, 48 neighbourhoods were selected (12 per city). Random start points were generated within each neighbourhood, and trained interviewers then approached every n^th^ house (determined by neighbourhood dwelling density) following a designated walk path for household enumeration and participant invitation to the study. A maximum of five visits were made to each eligible household. One adult per household was invited to participate, using next-birthday enumeration. Participant exclusion criteria were: outside the 20 to 65 year age range, not living in the household during the measurement period or for the 3 months prior, unable to speak English, or being walking-impaired.

### Protocol

Upon participant agreement, two home visits were scheduled. At the first home visit, informed consent was gained and participants were provided with an accelerometer to wear during waking hours over the next seven consecutive days (excluding water-based activities) alongside verbal and written instructions for accelerometer wear. Accelerometers were collected at the second home visit (approximately eight days later), at which time body size measures were taken and the study survey was administered by the interviewer.

### Measures

Body size: Height (m) to the nearest 0.1 cm and weight (kg) to the nearest 0.1 kg were assessed using a stadiometer and calibrated Seca scales, respectively. BMI was calculated as weight (kg)/height (m^2^). Waist circumference was measured at the mid-point between the iliac crest and the lateral costal margin to the nearest 0.1 cm using a Lufkin W606PM tape. International Society for the Advancement of Kinanthropometry protocols were followed for all body size measurements [40].

Built environment: GIS-derived spatial measures of the BE were generated as follows: (1) Dwelling density, number of dwellings/residential land area in a meshblock (per square kilometre); (2) Street connectivity, number of intersections with 3 or more intersecting streets per square kilometres within a meshblock; (3) Land use mix was calculated using the entropy index formula of Leslie et al. [41] (where k is the category of land use (residential, commercial/retail, public open space, industrial, other), p is the proportion of the land area in the specific land use (where the denominator is the total non-water/non-road area), and N is the number of land use categories), and (4) the ‘Neighbourhood Destination Accessiblity Index’, which is a measure of the intensity of neighbourhood destination opportunities within an 800 m buffer of the meshblock centroid [42]. Areas in water and roads were excluded from the area-denominator in all calculations. Neighbourhood level social deprivation was derived from the 2006 Census, providing area-level socioeconomic information at the meshblock level [43].

Streetscape: The Systematic Pedestrian and Cycling Environment Scan (SPACES) has demonstrated reliability for measuring environmental supports or barriers (e.g., safety, aesthetics) for walking and cycling [44]. This audit tool was modified for use in the New Zealand context [45] and implemented by trained fieldworkers. A total of 12 street segments were selected sequentially from a random start point in each neighbourhood. Fieldworkers audited each street segment (total *n* = 576) and individual street segment scores were combined for each neighbourhood.

Physical activity: Objective measurement of physical activity was captured using an Actical accelerometer worn above the right iliac crest on an elasticated belt (Mini-Mitter, Sunriver, OR). Units were set to collect data in 30 s epochs. At the end of the measurement period, data were downloaded within the manufacturer software, and raw accelerometer data were extracted from the Actical Export File (Version 02.10) listings for each participant. The raw data were read and plotted in SAS (version 9.2; SAS Institute, Cary, NC) for checking. Research officers were instructed to use event markers to record device delivery and collection so participants’ physical activity data were easily identified and extracted. Physical activity printouts were checked for excessive accelerometer counts (approximating >1000 counts per minute) and if identified, the accelerometer was considered faulty and that participants’ data removed from analyses (*n* = 26). Consequently, that day’s data and all successive data were set to missing. Data from participants engaged in shift work (*n* = 40) were also removed because timing of their daily activities and sleep varied from day to day. A conservative non-wear criterion of 60 consecutive minutes of 0 counts was employed [46] and these data excluded from analyses. Thereafter, any periods of less than 60 consecutive minutes during which the accelerometer was worn were also set to missing and these data excluded from analyses. This approach was employed to reduce the possibility of including data collected from movement of the accelerometer when not worn, or in case participants wore the accelerometer only while exercising. Cleaned data were reduced to mean number of accelerometer counts per hour while worn during weekend days and weekdays (weighted by individual wear-time). The count threshold for sedentary time was < 100 counts per minute [47].

Demographic information: The interviewer-administered survey captured information on respondents’ date of birth (to calculate age in years), sex, educational qualifications, marital status, employment status, household income, and car access. Combined household income before tax for the previous 12 months (in New Zealand Dollars, NZD) was assessed using seven categories; zero, <$20,000, $20,001 to $40,000, $40,001 to $60,000, $60,001 to $80,000, $80,001 to $100,000, and > $100,000. Data were later aggregated to five categories reflecting bands of $20,000; from < $20,000 to > $100,000 per annum. The median annual household income for New Zealand in 2010 was $75,700 [48].

Neighbourhood preference: Preference for a more suburban (less walkable) or urban (more walkable) neighbourhood (assuming housing cost, quality of schools, and mix of people were constant across neighbourhood type) was determined using survey items developed by Levine et al. [49], for which face validity has been established.

### Analyses

Significant correlations were observed across neighbourhood characteristics (Table 1); therefore each neighbourhood exposure was modeled individually with the two body size outcome measures (BMI, WC). To facilitate comparability across exposures, BE features were rescaled by dividing each feature by their SD calculated across all neighbourhoods.

**Table 1:** Correlation matrix showing associations between neighbourhood-level characteristics (*n* = 48 neighbourhoods)

	Dwelling density	Street connectivity	Mixed land use	NDAI	Streetscape	NZDep06
Dwelling density	1.00					
Street connectivity	0.89*	1.00				
Mixed land use	−0.02	0.09*	1.00			
NDAI	0.71*	0.75*	0.10*	1.00		
Streetscape	0.19*	0.26*	0.34*	0.39*	1.00	
NZDep06	0.36*	0.42*	0	0.38*	0.02	1.00

Note: *NDAI* Neighbourhood Destination Accessibility Index, *NZDep06* New Zealand Deprivation Index 2006

*p < 0.01

Accelerometer counts per hour, BMI, and WC were log-transformed to achieve approximately normal distributions. Non-linear relationships for the explanatory variables were investigated but none were found. Table 2 shows the correlations between outcome measures and neighbourhood level deprivation. Evidence suggests that physical activity and sedentary behaviours have distinct and independent effects on health [50, 51]; accordingly, these variables were considered in separate models as indicated below.

**Table 2:** Correlation matrix showing associations between outcome measures and neighbourhood-level deprivation (*n* = 48 neighbourhoods)

	Log-transformed accelerometer counts per hour	Percentage of time in Sedentary behaviour	Log-transformed waist circumference	Log-transformed BMI	NZDep06
Log-transformed accelerometer counts per hour	1.00				
Percentage of time in Sedentary behaviour	−0.72*	1.00			
Log-transformed waist circumference	−0.15*	0.13*	1.00		
Log-transformed BMI	−0.09*	0.10*	0.84*	1.00	
NZDep06	−0.05	0.03	0.11*	0.15*	1.00

Note: *NZDep06* New Zealand Deprivation Index 2006

*p < 0.01

Multi-level regression analyses were conducted in R using the package “lme4” [52] with random effects to account for the clustering of individuals within neighbourhoods (*n* = 48). Separate models were run to predict the associations between each BE measure with (a) BMI and (b) WC using three approaches as follows: Modelling approach 1 (hereafter termed Model 1 for simplicity) adjusted for sex, age, ethnicity, individual level socioeconomic factors (marital status, education, income, employment, car access), neighbourhood-level deprivation (theorized as a confounder and due to observed correlations with BE features and with body size – discussed in Results below), and neighbourhood preference (i.e., model fully adjusted for theorized and measured confounders to estimate total effect of BE on BMI); Approach 2 (hereafter termed Model 2) additionally included physical activity as a potential mediator (using the log of accelerometer counts per hour); and Approach 3 (hereafter termed Model 3) was identical to Model 2, with physical activity replaced by percentage of time spent in sedentary behaviour. Outcome and mediating models were created for the above models and mediation analysis was then performed using the “mediation” package in R [53, 54]. This was used to estimate the indirect effects between the independent variable (i.e., the BE variables) and body size (i.e., BMI and WC) via the mediating variables (i.e., physical activity and percentage of time spent in sedentary behaviour). A quasi-Bayesian approximation was used to calculate the *p*-value using 5000 Monte Carlo draws.

## Results

In total, 2033 adults participated (provided data from non-faulty accelerometers) in the study, reflecting a 44 % response rate. All data provided by the 2033 participants were included in the analysis to reduce bias introduced when decisions are made to include or exclude data [55]. Participants contributed a total of 16,869,826 counts per hour, averaging to 9,177 counts per hour across 1–7 days of data. Descriptive information for participants, outcome measures, and neighbourhood characteristics are provided in Table 3. The population sampled were predominantly female (57 %) and had at least some access to a car (93 %), consistent with the overall New Zealand population (51 % female [38], 92 % access to a motor vehicle [56]). Compared with the New Zealand adult population, a greater proportion were employed (86 versus 66 %) [57] and married (64 versus 45 %) [38]. A correlation matrix of BE features and neighbourhood-level deprivation is provided in Table 1. Statistically significant positive correlations were observed between neighbourhood-level deprivation and dwelling density, street connectivity, and destination accessibility (*r* = 0.36, 0.42, and 0.38, respectively, all *p* < 0.01). All BE features were significantly correlated with each other (*r* = 0.09 to 0.89, *p* < 0.01), with the exception of dwelling density and mixed land use.

**Table 3:** Participant and neighbourhood characteristics

Variable	Total^a^	Number	Percent
Age	2020		
15-29		450	22.3
30-44		782	38.7
45-54		462	22.9
55-65		326	16.1
Ethnicity	2033		
Māori		241	11.9
Non Māori		1792	88.1
Sex	2032		
Male		853	42.0
Female		1179	58.0
Qualification	2025		
No Qualification		546	27.0
School		234	11.6
Post School		474	23.4
Tertiary		771	38.1
Marital status	2028		
Never married		447	22.0
Married		1302	64.2
Previous married		279	13.8
Income ($NZ)	1821		
= <$ 40,000		421	23.1
$ 40.001-60,000		316	17.3
$ 60,001-80,000		271	14.8
$ 80,001-100,00		276	15.1
> $ 100,000		541	29.6
Employment	2030		
Full time		1182	58.2
Part time/Non-standard work		542	26.7
Unpaid		306	15.1
Car access	2032		
Un restricted		1633	80.4
Restricted		241	11.9
No car access		158	7.8
Neighbourhood preference	2006		
Strongly prefer walkable		700	34.9
Moderately prefer walkable		333	16.6
Neutral		286	14.3
Moderately prefer less walkable		207	10.3
Strongly prefer less walkable		480	23.9
Neighbourhood deprivation (NZDep06)	2033		
Least deprived quintile		419	20.6
		380	18.7
		419	20.6
		435	21.4
Most deprived quintile		380	18.7
Exposure and outcome variables	Total	Mean	SD
Body size			
BMI	2007	27.04	5.68
Waist circumference	1994	88.60	15.01
Accelerometer-derived measures	1838		
Physical activity (accelerometer counts per hour^b^)		9177	4803
Sedentariness (percentage of time sedentary)		57.49	11.89
Neighbourhood exposures^c^	2033		
Dwelling density		5.87	2.74
Street connectivity		5.42	2.39
Mixed land use		5.69	2.08
NDAI		11.74	4.93
Streetscape		87.57	11.26

Note: *BMI* body mass index (kg/m^2^), *n* number, *NDAI* Neighbourhood Destination Accessibility Index, *NZDep06* New Zealand Deprivation Index 2006, *$NZ* New Zealand dollars, *SD* standard deviation

^**a**^Data were missing as follows: Age, *n* = 13; sex, *n* = 1; qualifications, *n* = 8; marital status, *n* = 5; income, *n* = 212; employment status, *n* = 3; car access, *n* = 1; waist circumference, *n* = 39; BMI, *n* = 26; accelerometer data, *n* = 195

^**b**^Accelerometer units were counts per hour while worn (weighted by hours of data recorded)

^**c**^Means and standard deviations of neighbourhood exposures were calculated across study neighbourhoods

Values for correlations between outcome measures and neighbourhood-level deprivation are provided in Table 2. Correlations were all in the expected direction, with both body size measures being significantly correlated with each other (*r* = 0.84, *p* < 0.01), while a negative correlation was observed between physical activity and sedentary behaviour (*r* = −0.72, *p* < 0.01). Small but significant correlations were also observed between body size variables and sedentary behaviour (positive), neighbourhood-level deprivation (positive), and physical activity (negative).

The estimates for all associations with BMI and waist circumference for Models 1 to 3 are presented in Tables 4 and 5, respectively. In the fully adjusted model (accounting for individual characteristics and neighbourhood-level deprivation; Model 1), street connectivity, NDAI, and streetscape were significant predictors of BMI (a 1 SD increase in each was associated with between 1.27 and 1.41 % reductions in BMI, *p* < 0.05). For the same model, 1 SD increases in dwelling density, street connectivity, and NDAI were associated with reductions in waist circumference values of 1.97, 1.76 and 2.29 %, respectively (*p* < 0.05).

**Table 4:** Change in log-BMI for one SD increase in neighbourhood exposures (*n* = 1813)

Model	Neighbourhood exposure	Coefficient	(SE)	Indirect effect	*p*-value	Percentage of total effect explained
1 Adjusted for demographics, individual-level socioeconomic factors, neighbourhood-level deprivation, and neighbourhood preference	Dwelling density	−0.011036	0.005767		0.06	
	Street connectivity	−0.014172	0.005562		0.01*	
	Land use mix	−0.009173	0.005355		0.09	
	NDAI	−0.012825	0.005395		0.02*	
	Streetscape (SPACES)	−0.013417	0.004697		0.007*	
2 Plus including physical activity (log of accelerometer counts per hour)	Dwelling density	−0.009125	0.005768	−0.001924	<0.001*	n/a^a^
	Street connectivity	−0.012243	0.005598	−0.001889	<0.001*	13.2
	Land use mix	−0.008458	0.005297	−0.000741	0.17	6.9
	NDAI	−0.010952	0.005419	−0.00191	<0.001*	14.6
	Streetscape (SPACES)	−0.012626	0.004670	−0.000809	0.11	5.7
3 Model 1 plus including percentage time spent sedentary	Dwelling density	−0.0101833	0.0057573	−0.000873	0.18	7.3
	Street connectivity	−0.0131472	0.0055790	−0.001039	0.09	6.9
	Land use mix	−0.0089294	0.0053261	−0.00025	0.69	2.4
	NDAI	−0.0118039	0.0054092	−0.00109	0.06	8.1
	Streetscape (SPACES)	−0.0128561	0.0046980	−0.000570	0.31	4.1

Note: *BMI* body mass index, *CI* confidence interval, *n* number, *NDAI* Neighbourhood Destination Accessibility Index, *SD* standard deviation, *SPACES* Systematic Pedestrian and Cycling Environment Scan

*Significant at *p* < 0.05

^a^Inconsistent mediation – results not interpreted

**Table 5:** Change in log- waist circumference for one SD increase in neighbourhood exposures (*n* = 1801)

Model	Neighbourhood exposure	Coefficient	(SE)	Indirect effect	*p*-value	Percentage of total effect explained
1 Adjusted for demographics, individual-level socioeconomic factors, neighbourhood-level deprivation, and neighbourhood preference	Dwelling density	−0.019921	0.006521		0.004*	
	Street connectivity	−0.0177428	0.0066846		0.01*	
	Land use mix	−0.0020776	0.0067994		0.76	
	NDAI	−0.0231200	0.0059381		<0.001*	
	Streetscape (SPACES)	−0.0108457	0.0060160		0.08	
2 Plus including physical activity (log of accelerometer counts per hour)	Dwelling density	−0.017381	0.006388	−0.00248	<0.001*	12.3
	Street connectivity	−0.0151079	0.0065353	−0.00254	<0.001*	14.3
	Land use mix	−0.001062	0.006512	−0.000962	0.18	6.6
	NDAI	−0.0206553	0.0058506	−0.00242	<0.001*	10.4
	Streetscape (SPACES)	−0.0096708	0.0057933	−0.001126	0.08	9.6
3 Model 1 plus including percentage time spent sedentary	Dwelling density	−0.0191994	0.0063932	−0.000735	0.23	3.6
	Street connectivity	−0.0169189	0.0065567	−0.000822	0.17	4.5
	Land use mix	−0.0018313	0.0066369	−0.000214	0.71	1.9
	NDAI	−0.0223459	0.0058315	−0.000824	0.16	3.4
	Streetscape (SPACES)	−0.0102434	0.0058882	−0.000600	0.27	5.0

Note: *BMI* body mass index, *n* number, *CI* confidence interval, *NDAI* Neighbourhood Destination Accessibility Index, *SD* standard deviation, *SPACES* Systematic Pedestrian and Cycling Environment Scan

*Significant at *p* < 0.05

Adjusting for the potential mediation by physical activity and sedentary behaviour (Models 2 and 3, respectively) each compared to our ‘best’ total estimate of the BE-BMI association in Model 1 saw 13.2 % (street connectivity adjusting for time spent sedentary) to 14.6 % (NDAI adjusting for physical activity) of the BE-BMI association explained, and 10.4 % (NDAI adjusting for physical activity) to 14.3 % (street connectivity adjusting for physical activity) of the BE-WC association explained. An inconsistent mediation effect was observed for physical activity in the BMI-dwelling density relationship, that is, a non-significant association was found for the total effect, yet a significant mediating effect was found for physical activity. Therefore, no interpretation of results for this model have been provided [58].

## Discussion

Significant negative associations between objectively-assessed BE characteristics and body size were observed in the current study. After adjusting for individual characteristics, neighbourhood preference, and neighbourhood-level deprivation, street connectivity, and destination accessibility were associated with reduced BMI and waist circumference (ranging from −1.27 to −2.29 % for a 1 SD change in each BE variable). Higher quality streetscape, as assessed by the NZ-SPACES tool, was also associated with reduced BMI, but this relationship did not hold true for waist circumference. Dwelling density was associated with reduced WC (−1.97 %, *p* = 0.004), and this relationship neared significance for BMI (−1.10 %, *p* = 0.061).

When considering the results for the fully adjusted model, the differences found in body size may appear small. However, if we compare settings at either end of the BE spectrum, the consequences of living in these neighbourhoods on body size outcomes could be important. For example, when comparing the 5^th^ and 95^th^ percentiles of dwelling density in the current study (data reported in Witten et al. [18]), a SD of 3.14 is found. If the current study findings are applied to compare these two environments, a 3.14 SD in dwelling density would equate to a difference of approximately 1 BMI unit in an adult, or roughly 3–4 kg, dependent on height. It is important to note that observed associations are not independent of each other and that the approach taken to analysis in the current study does not enable conclusions to be drawn about the cumulative and combined effect of individual BE features on outcomes. For instance, if we consider street connectivity and destination accessibility, a number of scenarios may exist – one could be an indicator of the other, the two factors may interact, or they could have completely independent effects on body size; however it is not possible to determine which is the case from this study. With the exception of streetscape and neighbourhood-level deprivation, and mixed land use and dwelling density, significant correlations between all BE features were observed (*p* < 0.01), therefore collinearity was a concern.

The associations found between the BE and body size in the current study are consistent with earlier US research; when comparing the 90^th^ to the 10^th^ percentile for BE features in over 13,000 adults in New York City, Rundle et al. [59] observed reductions in BMI units of 0.41, 0.33, 0.34, and 2.86 kg/m^2^ with increases in mixed land use, bus stop density, subway stop density, and population density, respectively. Likewise, in a study of over 16,000 Texas adults, Hoehner et al. [30] determined that men and women living in neighbourhoods 1 SD above the mean for older homes (proxy measure of more walkable neighbourhoods) and shorter commute times had BMI values of 0.77 and 0.84 kg/m2 lower than those living in neighbourhoods 1 SD below the mean.

Advancing on this earlier work, the mediating effects of objectively assessed physical activity and sedentary behaviour on this relationship were considered. Consistent with earlier research [28, 32], no mediating effect was found for sedentary behaviour. While clear associations exist between sedentary time (particularly prolonged sitting) and body size in adults [60, 61], studies of associations between the BE and sedentary time are scarce [62]. It may be hypothesised from this small but consistent evidence base that a substantial relationship between the BE and objectively assessed sedentary time is unlikely. Conversely, it is also plausible that a clear understanding of this relationship has been limited by methodological issues, and lack of sensitivity and specificity in quantifying outcomes, particularly sedentary behaviours. For example, while improving on self-reported behaviours, the use of accelerometry to measure time spent sedentary is still imprecise; postural transitions cannot be assessed, and consensus is lacking on best practice for measurement protocols, data processing, cleaning, and aggregation [63]. We recommend employing additional methods in future to explore possible relationships between sedentary time and the BE in more detail (e.g., inclinometers, life-logging cameras, global positioning systems combined with accelerometry).

In contrast, a significant mediating effect of physical activity was observed on the relationship between body size and NDAI and street connectivity. These findings are consistent with the limited research that has considered the mediating effects of physical activity on the relationship between the BE and BMI [28–32, 64], and confirm the hypothesis that the relationship between BE and body size, mediated by physical activity, is not the result of systematic error. Preliminary analyses (not reported here) showed that the addition of neighbourhood preference to modelling resulted in no changes to relationships and only minor changes in the magnitude of differences. It is worth noting that when selecting their preferred neighbourhood, participants were requested to assume uniformity across neighbourhood types with respect to key issues such as schools, neighbourhood demographics and housing cost, which may have confounded findings somewhat as it is an idealistic measure. In addition, the neighbourhood preference measure employed was relatively broad, offering participants a choice of two neighbourhoods, essentially dichotomised by features that enable or disable use of active and public transport modes; consequently, selection effects may not have been captured completely.

The current study improves on limitations in previous research in this field [7, 65] by: undertaking robust assessment of the BE using contemporary GIS databases; purposefully selecting heterogeneous neighbourhoods in terms of walkability and region; randomly selecting participants; objectively assessing physical activity and body size (using BMI and waist circumference); using a continuous measure of BMI rather than thresholds (which can bias results [66]; a consideration especially important given the ethnic diversity in this study); adjusting for neighbourhood preference; and utilising mediation analyses to consider the effect of physical activity on the BE-body size relationship. Debate regarding approaches for accelerometer data reduction approach is ongoing [67], and to date, no agreed-upon best-practice method exists. In this study we employed a pragmatic accelerometer data reduction protocol that enabled inclusion of data for all complete hours of wear time (as opposed to limits of hours/days of wear), with the aim of reducing bias (for example with less compliant participants, [55]). Mean accelerometer counts were used to describe physical activity, recognising the issues surrounding the use of accelerometer count thresholds, and the contribution of activity of any intensity to health [68].

We did not estimate associations with individual BE characteristics with adjustments for all other BE features in the same model (due to collinearity) – this means that associations with each BE feature are not independent of associations with other BE attributes (as they are likely to be confounded by these variables). Caution should be applied when generalising these findings internationally, considering the substantial variability in urban form across countries [69]. It is also worth noting we did not consider the food environment in this study. In their earlier research, Brown et al. [29] undertook detailed and robust assessment of average caloric intake (two 24-h recalls); when controlling for this factor, a significant mediating effect of objectively-assessed physical activity still remained for the relationship between the BE and BMI. It is possible that clustering or collinearity exists between BE features that support physical activity and healthy nutritional practices; it is also plausible that the food environment (e.g., density of fast food outlets) may play an additive role with the physical activity environment in predicting body size.

While this study contributes to the cross-sectional evidence base, it does not enable the identification of causal relationships. Moreover, mediation analyses with cross-sectional data (particularly when stratified such as in the current study) can be subject to collider bias and measurement error [70]. Study findings are limited to New Zealand adults only; it is possible that differential findings may be observed for younger and older population groups, and for other population groups. With a response rate of 44 %, it is possible that self selection bias was present in the current study sample, although it is worth noting this response rate is higher than similar studies (e.g., 12 % in the study of Owen et al. [71], and 26 % in Frank et al. [37]).

Examination of relationships between objectively assessed body size, nutrition behaviours, physical activity, and food environments in future population research (particularly including younger and older participants) would be challenging, but an important next step in determining the relative contributions of behaviours and environmental features to obesity risk for people of all ages. Longitudinal, repeated measurements of BE exposures, risk behaviours, and body size outcomes are also necessary. Making use of existing longitudinal health datasets and alignign BE measurement with routine population studies are approaches that may make this feasible.

## Conclusion

This research contributes to the growing evidence base for links between the built environment and body size, and for the mediating effect of physical activity on this relationship. Longitudinal research, including objective outcome and environmental measures, across a range of demographically and geographically diverse population groups is needed to determine causality and generalisability of these relationships.

## Abbreviations

BMI: Body Mass Index
BRFSS: Behavioral risk factor surveillance system
GIS: Geographic information systems
IPEN: International Physical Activity and Environment Network
MB: Meshblock
NDAI: Neighbourhood Destinations Accessibility Index
NZ: New Zealand
SPACES: Systematic Pedestrian and Cycling Environment Scan
SD: standard deviation
WC: Waist circumference
URBAN: Understanding the Relationship between Activity and Neighbourhoods study
